# Does Foraging or the Avoidance of Predation Determine Habitat Selection by Selective Resident Grazers in the Serengeti Woodlands? A Mixed Strategy with Season

**DOI:** 10.3390/ani15152202

**Published:** 2025-07-26

**Authors:** Patrick Duncan, Anthony R. E. Sinclair

**Affiliations:** 1 UMR 7372 Centre d’Études Biologiques de Chizé, CNRS and Université de La Rochelle, 79360 Villiers-en-Bois, France; 2 Tanzania Wildlife Research Institute, Arusha P.O. Box 661, Tanzania; 3 Biodiversity Research Centre, University of British Columbia, Vancouver, BC V6T 1Z4, Canada

**Keywords:** ungulates, coexistence, processes, savanna

## Abstract

Savanna systems are characterised by a community of large mammal herbivores with up to 30 species; coexistence is based on resource partitioning. In this paper we analyse the features of the landscape and plant structure which lead herbivores to use particular locations, a key to resource partitioning. The processes involved, top-down versus bottom-up, are well known for large species and small ones but not for medium-sized ones. We use two resident, medium-sized species, topi (*Damaliscus lunatus jimela*) and kongoni, (*Alcelaphus buselaphus cokei*) in the central woodlands of the Serengeti; selection of habitat by the residents is important for predator-prey interactions and for interactions among the grazers. Using Principal Component Analysis and Multiple Regression we develop highly predictive models which show that resource availability is the critical determinant of habitat selection in the dry season; and reduction in predation risk appears to be important in the wet season. These results show for the first time that habitat selection by the medium-sized herbivores is driven by different strategies in the two seasons. This contributes to understanding the processes involved in the dynamics of this globally important savanna system, a necessary foundation for management.

## Simple Summary

Large herbivores play a central role in savanna ecosystems, structuring plants and feeding predators. As many as 30 species can coexist, from the large elephant to the small dik-dik. Coexistence is based on sharing resources but predation plays an important role for some species. Habitat selection is a key element of resource use: for the large species it is determined by resource availability, for the small ones the risk of predation plays a key role. The determinants of habitat selection by medium-sized species are not well known; the aim of this paper is to discover the features of the landscape and plant structure which determine their choice of habitat. Using detailed measurements of sward structure in the Serengeti we develop highly predictive models of habitat selection for two medium-sized species in the dry season, implying that foraging is the major driver of habitat selection. In the wet season reducing the risk of predation appears to play a dominant role, so these medium-sized species have mixed strategies. Since climate change will modify the resources and predator numbers are affected by disease outbreaks understanding the determinants of the strategies of the herbivores in this globally important system will contribute to effective models, an essential basis for any management actions.

## 1. Introduction

African savannas have a remarkable community of large herbivores, up to 30 coexisting species, with significant variability, regional and geographical (see the Kruger and Serengeti systems) and among habitats within the major systems (see [1,2]. Temporal variability is also significant especially in systems with migrant species (see [3]. Human impact of course imposes strong variability, both regional and temporal (e.g., [4]). Species coexistence is based on resource partitioning which is driven by spatial, temporal, or dietary differentiation, the key dimensions of which are the habitats used and the diets [1]. The herbivores eat different sets of plant species [5] and plant parts [6] and the determinants of diet selection, morphological and behavioural, are well understood [7,8].

For habitat use the patterns are clear: it has long been known that the herbivores move up and down the *catena* (footnote: Catenas are undulating hillslopes characterised by different soil types that create an environmental gradient from crest to bottom) in different ways according to their body size and season [9,10] and that the key resources in the dry season are located at the bottom of the catena [11]. In the Serengeti (Tanzania) foraging processes are the key determinant of habitat selection by the dominant herbivores, who migrate, these being wildebeest, *Connochaetes taurinus albojubatus*, plains zebra *Equus quagga boehmi*, and Thomson’s gazelle *Eudorcas thomsoni*, [12,13]. Other herbivores are resident in the central woodlands, an important part dry season habitat of the migrants, and among these buffalo (*Syncerus caffer caffer*), topi (*Damaliscus lunatus jimela*), and kongoni, (*Alcelaphus buselaphus cokei*), are key prey species for the predators. Selection of habitat by the residents contributes to ecological separation of the species and is also important for predator-prey interactions. Habitat selection by the large species is driven principally by food resources, while the small species are sensitive to the risk of predation. The behaviour of medium-sized species is less well understood; this paper focuses on the determinants of habitat selection by the resident, medium-sized selective grazers, topi and kongoni.

The populations of large ungulates are regulated by their food supply and those of small species by predators [14]. In the dry season the availability of food is the main determinant of habitat selection for the large species (buffalo, [15], see pages 56–62). Analysis of group associations has pointed to predator avoidance as being a driving factor for habitat selection by smaller species such as gazelles [16]. Anderson et al. [17], using camera-traps, analysed year-round associations of eight species of large grazers with habitat types by day and by night in the central woodlands of the Serengeti. They found that for the intermediate-sized species (topi and kongoni) habitat occupancy was also driven by predator avoidance. Therefore, predation risk may be an important determinant of where some ungulate species occur along the catena (predation hypothesis).

Habitat use by these ungulates is not just associated with grass structure and predation. Different water requirements among the species contribute to spatial and dietary niche differences at the scale of the Serengeti ecosystem [18]. However, at the fine scale of the study reported here (34 km^2^) water was available in rivers within 3 km so distance to water was not a determinant of habitat selection for the ungulates here.

Work in the Mara Reserve [16] and across the whole Serengeti system concluded that medium-sized species may have mixed strategies to cope with opposing pressures, balancing food availability and predation risk [19,20]. Similarly, in the Kruger ecosystem herbivores trade off food availability against the risk of predation in the wet season [21]. Hence, there is more than one possible mechanism underlying habitat selection by the medium-sized species. There are predictive models for selection of ‘hotspots’ by herbivores in the Serengeti in the wet season [19], but not for the dry season, when food is limiting. Here we use data collected more than 50 years ago because they are based on exceptionally detailed surveys of the structure of the swards and use these data to better understand the ecology of two little studied resident, medium-sized herbivores in an important habitat of the Serengeti, the central woodlands. We test the relative importance as driving factors of (1) the structure of the grass swards, or (2) the risk of predation. We used two perturbations as ‘experiments’, the switch between wet and dry season food supply, and secondly the passage of migrating wildebeest, zebra and Thomson’s gazelle. Both of these perturbations modify the food resources profoundly and so allow a number of mutually exclusive predictions from these hypotheses to be tested.

### 1.1. Food Hypothesis

The quantity of food is greatest near rivers and least at the top of the catena, especially in the dry season. If food abundance determines the choice of habitats on the catena, then the following hold:

(1) The resident ungulates do not stay in the same plant community and move down the catena towards the rivers in the dry season in order to select swards with large quantities of grass in view of their capacity to select high quality food from this type of sward (see [22] page 91, Figure 3.11 and [8]).
(2) In the wet season, grass is abundant everywhere, so the structure of the swards is less predictive of habitat selection than in the dry season and the relation is non-linear ([22] page 91, Figure 3.11).
(3) After passage through the study area by the migrants in the dry season, the quantity of food available is reduced. The residents continue to select habitats with larger quantities of grass. They move further down the catena compared to before the passage of the migrants to compensate for the change in quantity and structure of the swards.
(4) The habitats selected in the dry season differ in grass species from those selected in the wet due to their different location on the catena. Since the animals’ mechanisms of food selection (particularly their mouth sizes) are unchanged, the preferred swards in each season have similar structures.

### 1.2. Predation Hypothesis

Predation in the central woodlands of Serengeti is due principally to lions and the risk is greatest where the tree and thicket cover is moderate to dense, often along rivers, and in tall grass [20,23,24,25,26,27]. If predation risk is the principal determinant of habitat selection, then the following hold:

(1) The ungulates should select open vegetation types and avoid ones with thicker cover. They should avoid vegetation types with large quantities of grass in both seasons.
(2) In the wet season the risk of predation remains higher near thicker cover so ungulate distributions should be unchanged compared to the dry season.

## 2. Materials and Methods

### 2.1. Field Methods and Study Area

This work on habitat selection focuses on two resident and closely related selective feeders, topi and kongoni, between February 1971 and September 1973. Buffalo and the three migrants, wildebeest, zebra and the Thomson’s gazelle, were included when present in the study area. Topi and kongoni occurred here at similar densities (4.2 and 5.3 per km^2^, [28]). These populations remained stable between 1971 and 1989; topi were probably limited by predation and kongoni by competition for food [29].

The study area, 34.2 km^2^, was located south-east of Banagi Hill. The area is representative of the vegetation of the central woodlands [30], open *Vachellia-Commiphora* woodland dominated by the trees *Vachellia tortilis*, *Senegalia senegal*, *V. hockii*, *V. robusta*, *V. drepanolobium*, *Commiphora schimperi*, and by the medium height C4 grasses *Themeda triandra*, *Pennisetum mezianum*, *Chloris* spp., *Panicum coloratum*, *Eustachys paspaloides*, *Digitaria macroblephara*, and *Bothriochloa insculpta*. A landscape classification of the area ([28] and K.Gerresheim pers.comm.) used landscape units (facets) based on geology, soils and vegetation. Facets were grouped into the six main vegetation types (VTs) on the basis of the similarity of their vegetation (Table 1, Supplementary Materials Figure S1).

Each year of the study differed in the timing and intensity of the passage of migrants. In 1971 the passage was short but with high numbers—there were thousands of wildebeest in the study area for about a week (28 May–3 June); for the subsequent three months use by wildebeest, zebra and Thompson’s gazelle was light. In 1972 densities of wildebeest were much lower and the passage of the three migrant species was longer. Many zebras and gazelles with the wildebeest used the area for seven months, May-October.

Between February 1971 and September 1973, 18 monthly counts of the ungulates were conducted from a vehicle, between sunrise and sunset, with the positions of the animals noted on a vegetation map. For accuracy the counts usually took >8 h; there were fewer counts of the migrant species because they were usually absent in the wet seasons. Their densities were occasionally so high that they could not be counted accurately, however the relative numbers of migrants in the different vegetation types should be unbiased.

For 11 months the structure and quality of the vegetation was described on the day before or the day following the animal count, using detailed measurements of biomass, heights and proportions of leaf and stem (Table 2). These variables were chosen from a knowledge of quality of food eaten by ruminants [32], and detailed knowledge of the diets and feeding behaviour of the large herbivores in the Serengeti, including topi and kongoni (see [8,22,33]). In seven other months a restricted number of variables were measured: biomass of the herb layer, leaf height, culm height, and culm density.

Measurements were made at five sites in the two largest vegetation types (the Interfluve Ridges and Lower Plains, VTs 2 and 5 and in a third, the Drainage Lines (VT6) which was particularly important for the animals in the dry seasons. In the remaining three vegetation types (the Upper Plains, Rounded Hills and Open Grasslands, VTs 1, 3, and 4) three sites were used in each, making a total of 24 sites. The sites were chosen as the centre points of randomly selected hectare grid-squares, located using an aerial photograph.

At each site a transect of 25 step points was used to measure leaf and culm heights: the height of the closest leaf and culm to the toe of the shoe, was measured to the nearest centimetre. If more than one leaf was directly over the toe, the highest was measured. Mean culm density was estimated by throwing a 25 × 25 cm quadrat over the shoulder ten times and counting the number of culms rooted inside it, litter was not included. The same quadrat was thrown to provide four samples of clipped plants using shears: all herbaceous plants rooted within the square were clipped to ground level and weighed for biomass. The four samples (sometimes only two) were taken to the laboratory, dried at 70 °C for six hours and then reweighed, and manually sorted into forbs, green leaf, dead (dry) leaf and the stem plus sheath fractions of the grass. Forty-four green leaves of the dominant and of all the other species combined were measured. Confidence limits for the heights and lengths were low (5–15% of the means) but were higher for the other variables, culm density 10–30%, weights 10–30%, and composite variables 20–55%. Although these limits are relatively wide, they were sufficient to distinguish between the sward structures of the different vegetation types.

The principal predator in this area was the lion (*Panthera leo*). Offtake of prey by lions in the Serengeti was approximately twice that by hyaenas (*Crocuta crocuta*, see [34]). In the wet season buffalo, topi and kongoni were first, second and fourth most important prey species, and year-round they were third, fourth and sixth. There were about 50 lions in three prides around the study area ([20] Table 2, Figure 5, Table 43). Hyaenas were common south and west of the study area but were rare in the central woodlands, including the study area [35]. In the dry season some hyaenas followed the wildebeest, and a temporary den was found in the study area in 1972 but otherwise hyaena were not often seen or heard. Topi and kongoni constituted only a very small proportion of the diet of hyaenas (Mellina Sidous pers.comm.).

The risk of predation by lions in such woodlands is highest close to dense or moderately dense woody cover (see Introduction), the vegetation types are therefore classed qualitatively into three types, the two with the densest cover, the Lower Plains and Drainage Depressions (VTs 5 and 6) are classed as ‘Risky’, they are in drainage lines and often close to the rivers (see Table 1, and Supplementary Materials Figure S1). The Upper Plains and Rounded Hills (VTs 1 and 3) had less dense trees and bushes, so they are classed as ‘Intermediate’ and the Interfluve Ridges and Open Grasslands (VTs 2 and 4), which have sparse woody cover, ‘Less risky’.

### 2.2. Analysis

#### 2.2.1. The Seasons

The seasons in the Serengeti are usually defined with respect to rainfall, which generally provides enough moisture for grass growth between November and May but there are considerable variations among years [36,37,38]. After the first rains grass biomass increased; in the dry season biomass decreased depending on where rain and grazing occurred. For this study the seasons were defined on the basis of the rainfall and the biomass of grass in the Interfluve Ridges (VT2) the largest habitat, and which was strongly selected by the topi and kongoni during the wet seasons (see below, Supplementary Materials Table S2). In each year the grass biomass increased in December and fell sharply in May or August, Supplementary Materials Table S1. For this analysis the wet seasons were therefore started in December and the dry seasons started in June (1971) or in August (1972, 1973).

#### 2.2.2. Selectivity by the Ungulates

This was measured using Hunter’s Index [39], where the proportion of individuals of each species in a Vegetation type is divided by the proportion of the study area covered by that type. This index can vary from 0 to infinity with values > 1.0 indicating positive selection and values < 1.0 indicating avoidance; in practice the values for topi and kongoni fell between 0 and 8.0. There were usually 100–200 individuals of each species per count, fewer when the migrants were passing. Statistical significance was assessed using chi^2^ tests; the number of groups of a species in a habitat was used instead of number of individuals because individuals did not make independent decisions of choice. There were 10–30 groups of each species in the different counts. It was considered that strong selection for a habitat occurred when the index was statistically significant and its value > 1.5.

#### 2.2.3. Habitat Variables

Since sward variables were strongly interrelated the individual variables measured in the field could not be used in multiple regressions to analyse the processes involved in habitat selection. Principal Component Analysis (PCA) was used to describe the structure of the data in terms of a new set of variables (the Principal Components) conceptualised as underlying the original variables measured in the swards. PCA components summarise correlations between groups of interrelated variables in a quantitative way, so that where there are *p* variables, a smaller number, *m*, of Components may account for most of the variance. Secondly, the components themselves are orthogonal and can be used as independent, or predictive variables in regression analysis.

Principal Components (PCs) which accounted for >5% of the variance were used to develop prediction equations for habitat selection by each ungulate based on the structure and quality of the resources in the different habitats. The method used was stepwise Multiple regression [40]. The full set of sward variables was used for the dry seasons; for the wet seasons the restricted set of variables was used as the number of months when data were available was larger. Since the stony Rounded Hills, (VT3, 14% of the area) were selected only rarely this Vegetation type was not included in the modelling. When <30 individuals of a species were in the study area the count for this species was noted as missing for the modelling.

## 3. Results

### 3.1. Habitat Selection by the Selective Resident Grazers Along the Soil Catena

**Figure 1:**
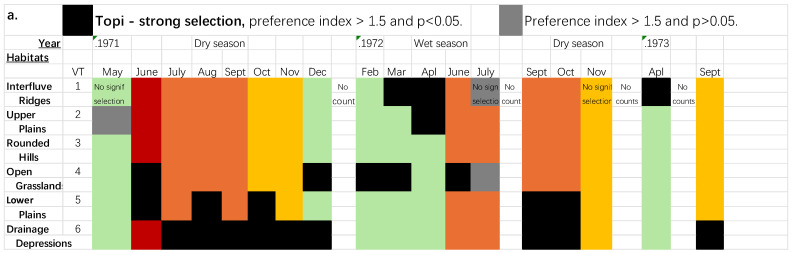

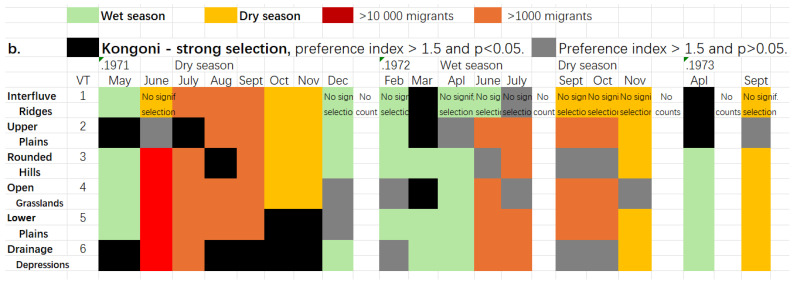
Habitat selection by (**a**) topi and (**b**) kongoni. For definition of the habitats (Vegetation types, VT1–VT6) see Table 1. Definition of the seasons see Supplementary Materials Table S1.

In the wet seasons both topi and kongoni selected Vegetation types in the upper part of the catena—Upper Plains and Interfluve Ridges (VTs 1 and 2, Figure 1 and Supplementary Materials Table S2) and Open Grasslands (VT4)—especially in the middle to late wet seasons.

In the dry seasons topi moved down the catena, selecting strongly the Lower Plains and Drainage Depressions (VTs 5 and 6, Figure 1 and Supplementary Materials Table S2). In the first year kongoni showed a very similar pattern of habitat selection to that of topi but were slower to move down the catena and they even selected the Rounded Hills which topi always avoided (VT3, Figure 1 and Supplementary Materials Table S2). In the second year there were many fewer kongoni groups than topi groups, their pattern of selection was similar to the previous year (Supplementary Materials Table S2), but statistically significant on only two occasions.

### 3.2. Plant Structure Along the Soil Catena 

The structures of the plant communities differed along the soil catena, the height of the grasses varying between short and medium, and the biomass of the swards from 15 g/m^2^ to 1000 g/m^2^. When data on the structure and quality of the swards (11 variables) are analysed across all VTs for both seasons combined, the strongest Principal Component, PC1 (‘Herb layer Quantity’), accounts for 86% of the variance in the data on sward structure: it has strong loadings on all the biomass and height variables (Supplementary Materials Table S3).

In the dry seasons the first Principal Component (PC1) is also a ‘Herb layer Quantity’ component (Table 3a). It has strong loadings on all the biomass and height variables and has a negative loading for ‘Green leaf’ proportions, i.e., the quality of the swards (the proportions of green leaf) is inversely related to grass quantity. PC1 accounts for 57.6% of the variance in the structure of the swards (Table 4a). The second Principal Component, PC2, ‘Stemminess’, has a strong loading for stem proportions and negative loadings for green and dry leaf proportions; this accounts for 24.1% of the variance. The third component, PC3 (‘Green leafiness’), accounts for 9.8% of the variance and has strong positive loadings for green leaf (both by percentage and weight, Table 3a) and negative loadings for percent dead leaf and culm height, expressing the fact that there were some leafy, green swards even in the dry season. PC4 has a strong positive loading for culm height; for green leaf the percent value is positive, but the weight of green leaf is neutral, indicating the presence of some VTs with ‘Tall swards with sparse green leaves’. These four components account for 97% of the variance in the structures of the dry season swards (Table 4a).

For the wet seasons the results were similar, but the importance of grass leaves was much greater than in the dry season (Supplementary Materials Table S3b). PC1 is again a ‘Herb layer Quantity’ component and accounts for 61% of the variance. PC2 is a ‘Green leaf’ component with large loadings, on the % green leaf and green leaf weight; PC3 has large loadings on the percentages of both green and dry leaf, so it is a ‘Total leafiness’ component. PC4 has large loadings on culm height and culm density, so it is a ‘Maturity’ component. These four components account for 96% of the variance in the wet season data on sward structures.

The Principal Component Analyses summarised effectively the detailed data on the structure of the swards in each season, so four PCs could be used to analyse how the structure if the swards influenced the movements of topi and kongoni up and down the catena. The main source of variance among the swards was the different quantities of grass; the other Components expressed variations in the quality of the swards and the accessibility of the leaves.

### 3.3. Plant Structure in the Swards Selected by the Ungulates

How do the different seasonal locations of the ungulates relate to the different sward structures in the habitats? In the dry seasons the prediction equations for habitat selection based on the structure of the swards account for 82% and 79%, respectively, for the kongoni and topi (Table 5). The most important component by far is the Herb layer Quantity component (PC1, Table 6) to which the selectivity of both species is positively related, with similar coefficients. Herb layer quantity alone accounts for over half the variance in the habitat preferences of the two species. They both also responded positively to the Green leafiness component (PC3, 17% and 8% of the variance, respectively, Table 5 and Table 6) and negatively to Tall swards with sparse green leaves (PC4, 11% and 7% of the variance, respectively). The sward structures and qualities, therefore, accounted for much of the habitat preferences of these two selective grazers. Overall, habitat use by topi and kongoni year-round was strongly correlated (r = 0.78, *n* = 120, *p* < 0.001); they reacted to the structure of the swards in similar ways. Nonetheless, the kongoni were less tied to the morphology of the catena than topi, selecting the stony Rounded Hills when migrants were present, topi rarely visited the Hills (Figure 1, Supplementary Materials Table S2); in the second dry season the kongoni pattern was similar but non-significant as only a few groups of stayed in the study area. The first prediction of the Food hypothesis can be accepted for both topi and kongoni.

Predictive equations for topi breeding groups in the dry season accounted for 92% of the variance in their habitat selection (Table 5), with two components contributing significantly (Table 6), namely Herb layer Quantity (PC1) and Green leafiness (PC3). For male topi (including territorial males and bachelor groups) the model accounts for less of the variance, 80% (Table 5); three components contributed significantly to the predictive equation for the males, positively for Quantity (PC1) and Green leafiness (PC3), and negatively for Tall swards with sparse green leaves (PC4).

**Figure 2:**
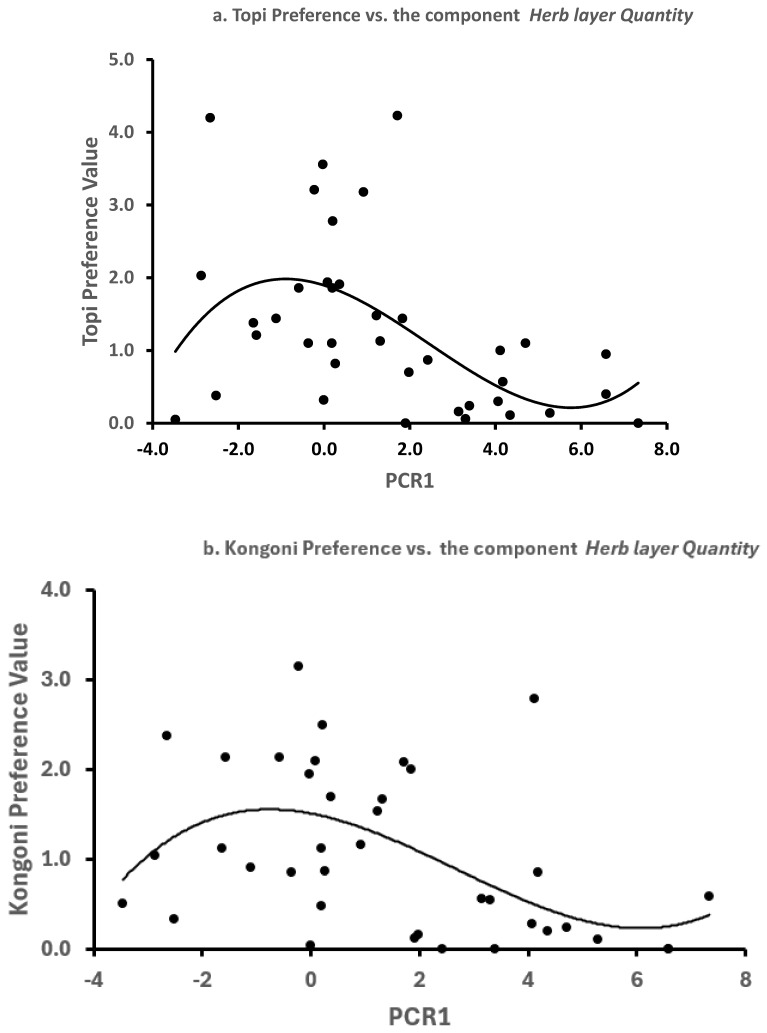
The relations between the values of the preference indices in the wet seasons and the scores of the Vegetation types on the Herb layer Quantity component (PCR1) of the restricted sward variables. The equations are in Table 5. (**a**) topi and (**b**) kongoni.

In the wet seasons the Herb layer Quantity component was the only PC which was significantly related to habitat selection and the relationship was non-linear (Figure 2, Table 5, Wet seasons). Topi and kongoni preferred swards of short to intermediate biomass, not the heaviest and tallest as in the dry season. These non-linear relations with the Herb layer Quantity component were much less predictive than the dry season models: for topi the proportions of variance in selection of the Vegetation types explained was 27% in the wet seasons and 79% in the dry seasons; for kongoni 25% was explained in the wet seasons and 82% in the dry seasons. Prediction 2 of the Food hypothesis stated that in the wet season the structure of the swards would be less predictive of habitat selection than in the dry season because grass is abundant everywhere: this prediction was supported for both species.

**Figure 3:**
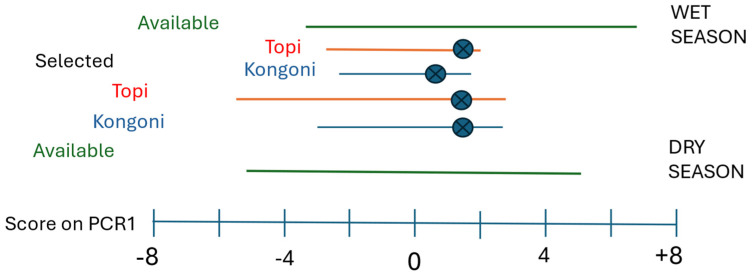
The available range of scores on the Quantity component (PC1) in the Wet and Dry seasons and the ranges selected strongly by topi and kongoni. The scores of the most strongly selected Vegetation types are indicated by 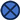.

Prediction 4 of the Food hypothesis leads to the expectation that the preferred habitats in the dry seasons would have structures similar to the preferred habitats in the wet seasons. The best summary of the differences in structure among the vegetation types is the scores on the Herb layer Quantity components (Supplementary Materials Table S3, PC1 and in the restricted set of variables PCR1, Table 3 and Table 4). In the wet seasons with large quantities of grass available, the scores of the available swards on Quantity, PCR1, ranged from −3.47 to +7.33 (Figure 3); topi showed strong preferences only for swards with intermediate grass biomass (with scores between −2.87 to +2.42) and kongoni were similar. The sward that was most strongly selected by topi had a score of +1.71 and that for kongoni was +0.20. In the dry seasons biomass available to the grazers overall was lower than that in the wet seasons, the scores on the Quantity component (PCR1) of the available swards ranged from −4.94 to +5.08. Topi showed strong preferences for swards with scores between −4.5 and +3.0; and the sward with the strongest preference had a score of +1.67. For kongoni it was the same Vegetation type on the same occasion as that for topi so the score of the most strongly selected Vegetation type was the same, +1.67. However, kongoni selected a much narrower range, never selecting swards with small quantities of grass.

The movements of these two species across the catena, therefore, meant that they used swards with very similar structures in both seasons. The Vegetation types where they found these swards were of course completely different botanically (Table 1). The seasonal movements up and down the catena, therefore, allowed topi and kongoni to stay in grass of similar structure, in spite of the profound changes between seasons in the available structures of all swards, in accordance with Prediction 4 of the Food Hypothesis.

The largest species, buffalo, also responded to variation in structure of the swards in the dry seasons, seeking habitats with large quantities of grass (i.e., high scores on the Quantity component PC1, Table 5 and Table 6). The prediction equation for buffalo accounted for 34% of the variations in their habitat preferences in the dry seasons (Table 6).

The migrants, wildebeest, zebra and gazelle, used the habitats ubiquitously, and the prediction equations for these species were barely, or not significant (Table 5), accounting for less than a quarter of the variations in these animals’ habitat preferences (13–24%). The three migrant species all preferred the habitats with short grass (i.e., with low scores on the Herb layer Quantity component PC1).

### 3.4. Impact of the Migrants on the Sward Choices of the Resident Selective Grazers

The passage of thousands of wildebeest migrating through the study area during one week in May–June 1971 was intense, with approximately 750 wildebeest/km^2^. They used all the Vegetation types and had powerful effects on the swards (Table 7a); biomass of the herb layer declined by 86% in the short grass swards and 76% in the medium height swards. In the following year the densities of wildebeest were lower, and biomass declined overall by 61%. The grasses became much less leafy (Table 7b), with reductions in the Leaf/Stem + sheath ratios of 68% and 78% in the two years, respectively.

After the passage of the migrants in 1971 topi moved further down the catena, showing strong selection for the Open Grasslands and Lower Plains (VT4 and VT5) in June (Figure 1, Supplementary Materials Table S2), and moved to the Drainage Depressions (VT6) at the bottom of the catena in July and for the rest of the dry season; the presence of large numbers of Thompson’s gazelles kept the grass very short in the upper parts of the catena, and hence unsuitable for topi. In 1972 the passage was different, there were fewer wildebeest in the study area, and, in contrast to 1971 when the swards were dry until October, in 1972 it rained heavily in May and June (102 and 132 mm, respectively). As a consequence, the grass biomass in June and July was greater than in 1971 (Supplementary Materials Table S1). During the long passage of the migrants (June–October 1972) topi again moved down the catena from VTs 1, 2 and 4 in June–July to VTs 5 and 6 in September-October (Figure 1). Kongoni showed the same sequence of movement as that of topi, but it was rarely statistically significant because only a few kongoni groups stayed in the study area. In addition, kongoni selected Rounded Hills (VT3), unlike topi. The third prediction of the Food Hypothesis can therefore be accepted for both topi and kongoni, since they moved further down the catena compared to before the passage of the migrants.

Since the topi and kongoni showed marked changes in their habitat selection in the presence of the migrants, and they followed the structure of the swards closely, selecting grass of similar structure in both seasons in spite of the strong effects of the migrants, as proposed by Prediction 3 of the Food Hypothesis, we conclude that all four predictions of this Hypothesis can be accepted for topi and kongoni.

### 3.5. Predation Risk and Habitat Selection

In the wet seasons, when food was abundant, topi and kongoni selected the habitats with lower risks of predation, namely Interfluve Ridges and Open Grasslands (VT2 and VT4, Table 1). These associations were strong and significant for nearly half of the time (Figure 1). Both topi and kongoni avoided the habitats with large quantities of grass (Figure 2). These results are in accordance with the first prediction of the Predation Hypothesis. In the dry seasons (June-November 1971, September-November 1972 and September 1973) the pattern of habitat selection changed profoundly, with topi strongly selecting the riskiest habitats, the Lower Plains and Drainage Depressions (VT5 and VT6), and also the habitats with the largest quantities of grass. The less risky habitats, the Interfluve Ridges (VT2) were never selected strongly by topi and the Open Grasslands (VT4) were selected only rarely (2/10 months, Figure 1., Supplementary Materials Table S2). Kongoni showed a similar pattern (Supplementary Materials Table S2), but their habitat selection was rarely statistically significant (Figure 1). Both predictions of the Predation Hypothesis can be rejected for both species in the dry seasons.

These data covered the daytime only. A topi group was followed in both seasons, for nine nights over the two years; for eight of these nights, they stayed in a single small part of their territory, about 5 ha in Open grassland (VT4) with a few small trees (*Vachellia drepanolobium*), which provided very little cover for predators. On the other night in May 1973, they stayed in the Interfluve Ridges (VT1) where they had been feeding during the day. The cover there was thicker, with patches of dense *Senegalia mellifera,* and they were stalked by a lion. The following night they returned to their usual nocturnal resting place and spent the night there ([22] Figure 6.3). Hence, the little information available suggests that these topi generally spend their nights in the less risky Open Grassland, year-round, in contrast to the seasonal changes in their use of habitats during the daytime.

### 3.6. Abundance of Topi and Kongoni—Impact of the Migrants

During the wet season, when the migrants were absent from the study area the numbers of topi varied between 132 and 192 and kongoni between 106 and 213 (Table 8). In the days after the passage of the wildebeest in June 1971 the numbers of topi and kongoni declined to 32 and 18 individuals, respectively, including only three female kongoni. The numbers of topi returned almost to their wet season values after two months, with a median number of 121 during the four months when the migration was in the area (June–September, 88% of the wet season numbers). Kongoni numbers remained low, with a median number of 71 during these four months (40% of the wet season numbers). In 1972 the pattern of the migration was different with wildebeest, and particularly zebra and gazelles, using the area for seven months, April–October, and never reaching such high densities as in 1971. During these seven months the numbers of topi (median 128, 74% of the wet season numbers, range 114–149, Table 8) and kongoni declined (median 85, 54% of the wet season numbers, range 25–205).

## 4. Discussion

### 4.1. Habitat Selection by Resident, Selective Grazers in Savanna Habitats; The Processes

Among the many medium-sized ungulates in these communities, topi and kongoni were chosen for this study because detailed work on their feeding behaviour allowed appropriate methods of describing the structure of the swards to be used, and appropriate hypotheses to be developed on the habitats they would use preferentially (see 1. Introduction second paragraph and 2. Materials and Methods, fifth paragraph). They are representative of grazers but not necessarily of mixed feeders.

In this study the topi and kongoni moved up and down the catena in ways which are consistent with previous work [10]. They usually selected the upper parts of the catena strongly in the wet seasons, and the lower parts in the dry seasons, but the pattern of seasonal changes was neither constant between years, nor clearly related to topography in any simple way. Water was always available within 3 km, so at the scale of this study this resource was not an important determinant of habitat use here.

In the wet seasons both species preferred habitats with less risk from lion predation (Open Grasslands and Upper Plains, Figure 1, Supplementary Materials Table S2) so evading predation may have influenced habitat selection when food was abundant. However, both species also selected the riskier Interfluve Ridges at the top of the catena in the wet seasons. So the structure of the swards is likely to have also influenced their choices in this season, though to a smaller extent because grass abundance accounted for only 27% (topi) and 25% (kongoni) of the variance in the wet season choices (Figure 2). Bukombe et al. [42] found similar behaviour in the western corridor of the Serengeti, where topi preferred Grassland and avoided more risky vegetation types in the wet season but switched to risky woodland types in the dry season. In Hwange Park, Zimbabwe, when lions were nearby, ungulates also selected open areas ([43], see also [44]).

At night, when there is a greater risk of predation, topi used an open habitat, which was less risky. Kongoni moved to short grasslands at night in Nairobi National Park, Kenya [45] and when resting during the day [46]. Male topi also used short grassland when lekking, apparently to reduce the risk of predation [47]. Zebra also use open habitats more at night in the Kruger National Park [21]. The effect of the day/night cycle on habitat selection, therefore, appears to be fundamental; these differences as well as the different requirements of animals of different ages and sexes need further work.

In the dry seasons the two species moved down the catena maintaining themselves in swards with similar structures to those preferred in the wet season (Figure 3) and were clearly strongly influenced by the impact of the migrants on their food resources (Figure 1) rather than simply staying in the less risky habitats. Overall, the habitat choices made by the topi and kongoni were in accord with the Food Hypothesis, but in the wet season their choices appear to be driven by both Hypotheses. Topi and kongoni therefore have mixed strategies of habitat selection among seasons and probably between day and night.

Detailed experimental work has shown that topi and kongoni have very similar foraging behaviour and both extract diets of high quality from taller swards, with later growth stages than wildebeest. Topi specialise on green growth at an intermediate or mature stage ([22] pages 91–2), and grass quantity has a positive effect on their obtaining a good quality diet in dry conditions; in wet conditions grass quantity is again the key but has a non-linear effect. Kongoni specialise on mature growth stages of grass, even where the sward is dry and senescent [8]. The habitats these selective feeders sought (where the grass was of intermediate height and relatively leafy) are consistent with their foraging behaviour. In contrast, wildebeest have faster bite rates and higher rates of food intake on early growth stages than either topi or kongoni, specialising on short grass [8], which corresponds with the predictive model for their habitat selection (Table 5).

The Isolated rounded hills (VT3) were not included in the modelling. These were almost always avoided by all species: this may be due to the energy needed to climb up and down the hills. Landscape as well as the food resources may therefore affect habitat selection, particularly by topi.

These results are consistent with work at broader geographical and temporal scales: topi and kongoni overlap extensively in their habitat selection in the central woodlands and have mixed strategies ([20] Figure 4b,c). Hopcraft et al. [20], covering the whole ecosystem south of the Mara River, showed that in the wet season all the resident ungulates selected open habitats with good food quality and reduced risk of predation. At this scale, they concluded that intermediate sized grazers (kongoni and topi) seek grass biomass of sufficient quality in relatively predator-safe areas, even during diurnal hours. A detailed, fine grained study of habitat selection in the Kruger system Burkepile et al. [21] also found trade-offs between food acquisition and minimising predation risk for two grazers, wildebeest and buffalo in the wet season, so the conclusions reported here appear to be generalizable for wet season conditions in the Serengeti and beyond. We look forward to seeing the results of new field research with detailed measures of sward structures in dry seasons. Anderson et al. [17,19] showed from camera-traps (covering the day and night, over the whole year) that food abundance influenced habitat choice by resident buffalo and migrant zebra, wildebeest and gazelle. Habitat occupancy by intermediate-sized topi and kongoni, as well as small gazelles, appeared to be driven by predator avoidance. In the study of diurnal habitat selection reported here, predator avoidance was found in the wet season, but not in the dry.

In another study on the floodplains of the western Serengeti topi used four Vegetation Types, in the wet season preferring the upper two, Short grass and Medium grass, and in the dry season the lower two, Long grass and Very long grass. In the analysis of habitat selection, the dry season model explained 87% of the variance (*p* < 0.001, [22] page 114 and Table 4.8) and the main variable was PC1 which accounted for 77% of the variance. In the wet season analysis, preference for the Sward quantity (PC1), was of a very similar hyperbolic shape to the model in the woodlands reported here and accounted for 31% of the variance ([22] pages 113–4 and Table 4.8). On the floodplain, where there was little predation by lions and hyaenas, other sward variables came into play and the wet season model accounted for 83% of the variance in habitat selection by topi.

The comparison of resource use in the dry and wet seasons is a necessary step. At the time of the present study more subtle distinctions were not recognised. To nuance the understanding of the animals’ strategies fine-grained data should be collected to understand how avoidance of dense cover varies across different vegetation types and how microhabitat features override the general pattern. We have shown that animals of different sexes have different strategies; it will be important to deepen this by obtaining more fine-grained data on how individuals use resources differently according to their age, sex, reproductive and social status (in relation to calving, mating, territorial, bachelor status, etc.). Reproduction leads to profound changes in the behaviour especially in topi where calves lie out alone in their first weeks, males seek territories on leks, and females prefer to mate with particular males. Analysing how resource use by adults and young is affected by lying out, rutting and by oestrus would deepen our current understanding considerably; this is now feasible with modern technology, in particular satellite imagery and tracking with GPS.

Kongoni were more ubiquitous than topi in their choice of habitats and were more sensitive to the passage of the migration (Table 8). However, the two species showed very similar patterns of feeding and habitat use (Figure 1), so they show very little niche separation which poses the questions of functional redundancy and mechanisms of coexistence. They are so similar that they may show functional redundancy where they overlap. They can coexist because their distributions in the Serengeti (and continentally) are quite different: kongoni are centred on the woodlands and the dry east of the Serengeti, while topi occur at high densities in the wetter western floodplains (see [48], Figure 4.10B). In the floodplains only two kongoni were seen in three years of fieldwork (P. Duncan pers.obs.). In the Mara Reserve kongoni occurred at low densities where topi occurred at high densities [49]. Overall topi preferred areas with high rainfall and richer soils ([48] Figure 4.11). In contrast, kongoni preferred low to medium rainfall areas, around 700 mm/year and did not select high nutrient areas ([48] Figure 4.11). These strongly different distributions at the regional and continental scales are likely to reflect the ecological and evolutionary contrasts between the two species. Kongoni have

- lower water requirements than topi [18] and
- lower levels of food intake [8].

The result of these ecological contrasts is that kongoni have ‘slow’ demographics compared to topi, with slow growth and low breeding rates [22,45] and higher survival in semi-arid areas. In wetlands with their higher levels of nutrients topi have high growth rates, shorter gestation times and breed at faster rates.

### 4.2. Impact of the Migrants on the Abundance of the Resident Selective Grazers

The three migrant species used the six Vegetation types much less selectively than the resident grazers, and they reduced the food available to the residents. Consequently, in 1971 numbers of topi and kongoni declined by over two thirds after the week-long passage of the wildebeest. Only three female kongoni remained, compared to over 50 before this passage. Most of the remaining kongoni were single males, probably territorial. The numbers of topi returned to previous levels after a few weeks, but those of kongoni only did so after rains had allowed the grass to grow. This difference between the two selective feeders fits with the fact that topi can maintain adequate rates of intake on grass as short as 3 cm [50], while kongoni have lower intake rates on all swards ([8] Figure 2). In conclusion, the impact of the migrant wildebeest on the residents was strong and could limit resident population numbers if food is limiting.

More information is needed on the processes driving selection of habitat by these ungulates, in particular habitat use needs to be compared between day and night. Burkpile et al. [21] consider that “…limiting observations to daylight hours may miss the strongest signal of predation risk on habitat selection.” Further, feeding in risky habitats may impose costs on the herbivores: behavioural observations are needed, for instance to test if herbivores spend more time alert and less time feeding in risky than in safer habitats, or whether variations in predator density or activity across seasons alter ungulate avoidance behaviour as observed by Fitzgibbon and Lazarus [51] for Thomson’s gazelle.

The aim of this study was to deepen understanding of a critical element of the dynamics of savanna systems, why herbivores select certain habitats, since this selection determines where the herbivores have most impact on plants and nutrient cycles; habitat selection also of course influences predator-prey interactions strongly. The approach used here may contribute to management through predictive modelling. Holt et al. [3] consider that “Rules by which herbivores move towards more rewarding food patches play a major role in determining their dynamics. How these considerations alter interactions that involve predation remains to be explored.”

## 5. Conclusions

In the dry season when food is limited the food resources are the principal determinant of diurnal habitat choice by the selective feeders, topi and kongoni, with females being more selective than males. The choices made by these grazers correspond closely with their abilities to extract good quality diets from swards of varying structure. In the wet season when food was abundant the risk of predation appears to be an important determinant of habitat choice for these intermediate-sized grazers. This is likely to be true at night in all seasons, when the risk of predation is high. Topi and kongoni, therefore, had mixed strategies in the Serengeti woodlands, since the processes driving their choices of habitat varied according to the season. The effect of the migration in the dry season provided the conditions to test how a reduction in food altered the choice of habitats and food. An overall model still needs to be developed to predict habitat selection quantitatively by individuals, in both seasons, in different activities, by day and by night.

## Figures and Tables

**Table 1:** The Vegetation types in the study area, 34.2 km^2^; for a map see Supplementary Materials Figure S1.

Based on the Facets in a Landscape Classification of the Serengeti National Park ([31] and K. Gerresheim pers.comm.).						
Facet	Vegetation Type	Morphology	Common Woody Plants	Dominant Grasses	Area %	Predation Risk
9a	VT1	Upper part of wide, gently concave plains, very well drained	Mostly grassland with sparse, *Vachellia tortilis* and patches, of *Senegalia mellifera*	*Sporobolus marginatus*, *Digitaria macroblephara*, *S. pellucidus*, *Panicum coloratum*	11.7	Less risky
4, 5b	VT 2	Elongated interfluve ridges, gently rounded and very well drained	*V.robusta*, *V. tortilis*, *V. hockii*, *S. senegal*, *Commiphora, schimperae*	*Chloris roxburghiana*, *D. macroblephara*, *Bothriochloa insculpta*, *S. marginatus*	35.2	Intermediate
1b	VT 3	Isolated rounded hills	*V. hockii*, *S. senegal*,	*Eustachys paspaloides*, *Heteropogon contortus*	14.4	Intermediate
5, 5a			*Ormocarpum trichocarpum*	*S. fimbriatus*, *D. macroblephara*, *Themeda triandra*		
9b	VT 4	Middle concave plains, well drained	Open grassland with, some *V. drepanolobium*	*T. triandra*, *P. coloratum*, *Microchloa kunthii*, *S. festivus*	7.8	Less risky
9b	VT 5	Lower concave plains,	*V. drepanolobium*, some *V. tortilis*	*Cenchrus mezianus*, *T. triandra*, *P. coloratum*,	24.1	Risky
11, 13		fairly well drained		*B. insculpta*, *Cymbopogon excavatus*		
9c, 14a, 14b	VT 6	Shallow elongated drainage lines and minor valleys, ephemeral surface drainage.	*V. tortilis*, *V. robusta*, *Albizia harveyi*, some *V. seyal* and *Kigelia africana*	*T. triandra*, *P. mezianum*, *Chloris gayana*, *Sorghum verticilliflorum*, *P. coloratum*, *Echinochloa haploclada*	6.8	Risky

**Table 2:** The variables measured to describe the structure and quality of the swards.

*Restricted variables—four were measured in 18 months:*
Biomass of the herb layer, leaf height, culm height and culm density.
*Nine variables were measured in 11 of the 18 months:*
Weight of grass green leaf
Weight of grass dry leaf
Weight of grass stem and sheath
Length of leaves of the dominant grass species
Length of leaves of the other grass species.
From these three composite variables were calculated:
% green leaf in the sward
% dry leaf in the sward
% stem and sheath in the sward

**Table 3:** The loadings of the sward variables on the four main Principal Components (PC) in the two seasons used for the predictive models of habitat selection by the ungulates.

a. Dry seasons				
Principal Component	PC1	PC2	PC3	PC4
Green leaf %	−0.385	−0.667	0.541	0.333
Dry leaf %	0.237	−0.831	−0.472	−0.173
Stem %	0.000	0.993	0.111	−0.026
Sward biomass (log)	0.969	0.071	0.055	−0.131
Leaf height (log)	0.929	−0.009	0.032	0.251
Culm height (log)	0.704	0.191	−0.454	0.478
Culm density (log)	0.952	−0.038	−0.013	0.103
Green leaf biomass (log)	0.826	−0.257	0.444	−0.010
Dry leaf biomass (log)	0.908	−0.303	0.014	−0.239
Stem biomass (log)	0.918	0.292	0.206	−0.159
b. Wet seasons				
Principal Component	PCR1	PCR2		
Sward biomass (log)	0.960	−0.02		
Leaf height (log)	0.897	−0.37		
Culm height (log)	0.960	−0.01		
Culm density (log)	0.889	0.429		

**Table 4:** The proportion of the variance in the structure of the swards of the six Vegetation types accounted for by the first four Principal Components. See Table 2 for the variables analysed.

a. Dry Seasons—from the Full Analysis of the Swards.	
Principal Component	% of variance
PC1	57.6
PC2	24.1
PC3	9.8
PC4	5.4
Total	96.9
b. Wet seasons—from the analysis of the restricted set of sward variables.	
Principal Component	% of variance
PCR1	86.4
PCR2	8.0
PCR3	3.5
PCR4	2.1
Total	100.0

**Table 5:** Prediction equations for habitat selection by ungulates in the Serengeti woodlands in the dry and wet seasons.

PC1, PC2, PC3, PC4—The Scores on the First Four Principal Components.			
Species	Dry Seasons	Variance Explained (%)	Significance (p)
Kongoni	Preference = 1.26 + 0.189 PC1 + 0.625 PC3 − 0.886 PC4	82	<0.001
Topi (all)	Preference = 1.23 + 0.214 PC1 − 0.803 PC4 + 0.498 PC3	79	<0.001
Topi (breeding)	Preference = 1.30 + 0.294 PC1 + 0.686 PC3	92	<0.001
Topi (males)	Preference = 1.29 + 0.188 PC1 − 0.680 PC4 + 0.743 PC3	80	<0.001
Buffalo	Preference = 1.23 + 0.383 PC1	34	<0.01
Zebra	Preference = 0.854 − 0.065 PC1	24	<0.05
Wildebeest	Preference = 0.959 − 0.053 PC1	13	<0.10
Thomson’s gazelle	Preference = 0.966 − 0.117 PC1	16	<0.10
**Prediction equations for habitat selection in the wet seasons.**			
PCR1—The score on the first Principal Component (‘Herb layer Quantity’) from the analysis of the restricted set of sward variables.			
Topi (all)	Preference = 1.90 − 0.186 PCR1 − 0.0875 PCR1^2^ + 0.012 PCR1^3^	27	<0.01
Kongoni	Preference = 1.51 − 0.115 PCR1 − 0.0657 PCR1^2^ + 0.0082 PCR1^3^	25	<0.01

**Table 6:** Proportion of the variance in habitat selection by ungulates accounted for by the scores of the first four Principal Components in the Serengeti woodlands in the dry seasons.

**Species**	**Component**	**Variance Explained (%)**
Kongoni	PC1	54
	PC3	17
	PC4	11
Topi (all)	PC1	64
	PC3	8
	PC4	7
Topi (breeding)	PC1	79
	PC3	13
Topi (males)	PC1	44
	PC3	24
	PC4	12
Buffalo	PC1	34
Zebra	PC1	24
Wildebeest	PC1	13
Thomson’s gazelle	PC1	16

**Table 7:** The impact of the migration on the swards, (a) biomass and (b) leafiness. Data from this study, or for the western plains from [41].

**a. On the Biomass**												
		Short grass swards		**g/m^2^**			Medium height swards			**g/m^2^**		
		Before	95% cl	After	95% cl	Reduction %		Before	95% cl	After	95% cl	Reduction %
Woodlands	1971	110	25	15	9	**86.4**		170	21	40	15	**76.5**
							All swards	Before				
Woodlands	1972							195	31	75	30	**61.5**
							Medium height swards					
Western plains	1974							457		69		**84.9**
**b. On the leafiness of the grass**												
							All swards		**Leaf/Stem + Sheath ratio**			
Woodlands	1971							Before		After		Reduction %
								1.23		0.4		**67.5**
Woodlands	1972							Before		After		
								1.5		0.33		**78.0**

**Table 8:** Effect of the wildebeest-led migration on the numbers of topi and kongoni in the study area. The seasons were defined from the rainfall and the biomass available in the herb layer, see Supplementary Materials Table S1. The migration is indicated: in red when there were >10,000 wildebeest; in brown when >1000 migrants.

**1971**	**Topi**	**Kongoni**		**Rain mm, Monthly**	**Season**	**Grass Biomass (g/m^2^)**
13, 18 February	188, 136	189, 175		36	‘Wet’	Not measured
March	140	176		23	‘’	Not measured
May	132	172		188	‘’	173
1st June	40	32	c. 25 000 wildebeest		‘Dry’	Not measured
11 June	89	71	c.100 Wbst, c.2600 Zebra, >100 Gazelles.	2	‘’	50
July	129, 129	62, 83	On 23rd c.200 Wbst, c.90 Zebra. >100 Gazelles both dates.	49	‘’	Not measured
15, 23 August	57, 114	31, 74	On 15th no Wbst or Zebra, hundreds of Gazelles & Buffalo. on **23rd** no Wbst, <50 Zebes in gps of <10, hundreds of TG, no Buff.	25	‘’	Not measured
3 September	150	144	>2000 Wbst,>1000 Gazelles, >200 Zebras.		‘’	Not measured
24 September	139	71	<100 Wbst,>1000 Gazelles, 4 Zebras, 250 Buffalo.	8	‘’	Not measured
25 October	162	135	No Wbst, >100 Gazelles, <10 Zebra, 250 Buffalo.	101	‘’	66
29 November	105	69	No Wbst, Gazelles; >100 Zebra.	57	‘Dry’	51
December	174	156	No Wbst, <20 Gazelles; 145 Zebra, 18 Buffalo.	52	‘Wet’	92
**1972**						
January	No count	No count		23	‘Wet’	Not measured
February	162	106	No Wbst, 6 Gazelles; c. 100 Zebra.	143	‘’	101
March	192	213	No migrants	76	‘’	131
April	128	110	Zebra moving thro, Wbst in smallish herds, <500	72	‘’	104
May	No count	No count	Zebra moving thro, then Wbst, strong impact on grass.	108	‘’	62
8 June	114	25	500 Wbst, tens of zebras, no Gazelles.	132	‘’	78
2nd July	129	205	No Wbst, 2000 Zebra, 5 Gazelles, 380 Buffalo.	0	‘’	98
August	No count	No count		72	‘Dry’	31
9 September	79	85	c. 400 Wbst, c.800 Zebra, c. 1000 Gazelles	86	‘’	57
October	149	70	c. 1700 Wbst, c.250 Zebra, c. 350 Gazelles, 31 Buffalo.	56	‘’	57
23 November	223	214	6 Wbst, c.350 Zebra, no Gazelles, 49 Buffalo.	185	‘’	64
December	No count	No count		206	‘Wet’	102
**1973**	No count	No count				
January	No count	No count		79		Not measured
February	No count	No count		72		Not measured
March	No count	No count		10		Not measured
27 April	178	204	c. 500 Wbst, c.2000 Zebra, c. 1000 Gazelles	49	‘Wet’	116
May	No count	No count		83		Not measured
June	No count	No count		24		Not measured
July	No count	No count		0		Not measured
August	No count	No count		20		Not measured
18 September	241	210	c. 700 Wbst, c.400 Zebra, c. 600 Buffalo, c.350 Gazelles	175	‘Dry’	68

## Data Availability

The information on selection of habitats by topi and kongoni analysed in this paper is given in Supplementary Materials Table S2; the field data on habitat selection and the sward structures can be obtained on request to PD.

## Supplementary Materials

The following supporting information can be downloaded at: https://www.mdpi.com/article/10.3390/ani15152202/s1, Figure S1: Appendix 1. The woodland study area showing the distribution of the Vegetation types.; Table S1: The seasons in this analysis.; Table S2: Habitat preferences of topi and kongoni.; Table S3: The matrices of the loadings of the variables on the four main Principal Components (PCs). a. Over all months b. For the wet seasons.
