# Supplementary figures and images for: Does Foraging or the Avoidance of Predation Determine Habitat Selection by Selective Resident Grazers in the Serengeti Woodlands? A Mixed Strategy with Season
Source: Animals (Basel). 2025 Jul 26;15(15):2202. doi: 10.3390/ani15152202 (PMC12345477; doi:10.3390/ani15152202)

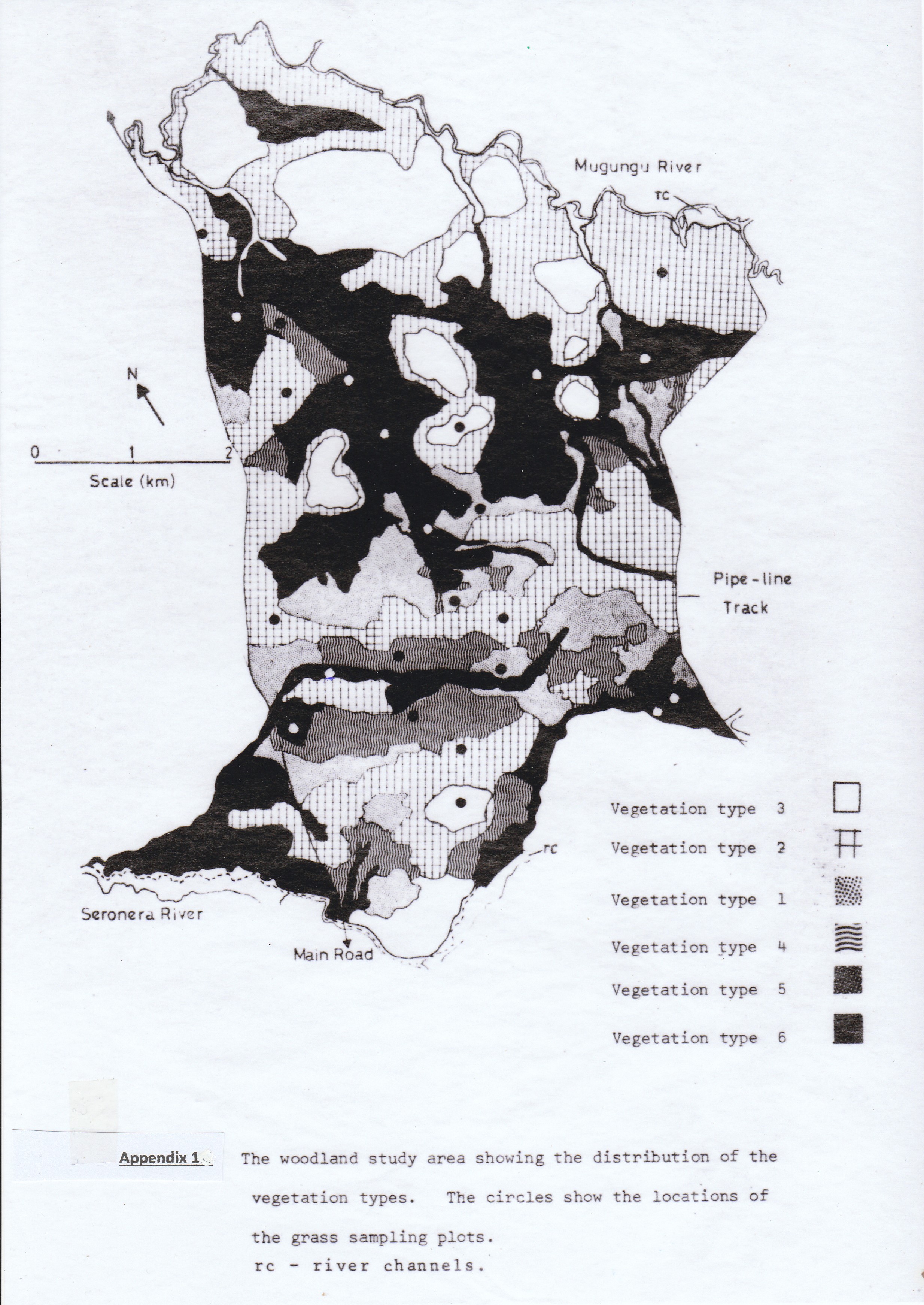

Supplement: Supplementary file 1 [file animals-15-02202-s001.zip › Figure S1..jpg]
